# Non-Conventional Brewers’ Spent Grains, an Alternative Raw Material in Bread-Making

**DOI:** 10.3390/foods13213442

**Published:** 2024-10-28

**Authors:** Mariana-Liliana Păcală, Alexandrina Sîrbu, Anca Șipoș

**Affiliations:** 1 Faculty of Agricultural Sciences, Food Industry and Environmental Protection, “Lucian Blaga” University of Sibiu, 550024 Sibiu, Romania; mariana.pacala@ulbsibiu.ro; 2 FMMAE Ramnicu Valcea, “Constantin Brancoveanu” University of Pitesti, 240210 Ramnicu Valcea, Romania; 3 Doctoral School-Plant and Animal Resources Engineering, University of Life Sciences “King Mihai I” from Timișoara, 300645 Timișoara, Romania

**Keywords:** brewers’ spent grain (BSG), by-product, buckwheat, oats, non-conventional, fermentation, sourdough, BSG upcycling, functional bread

## Abstract

The main objective of this experiment was to investigate the technological potential of upcycling unsparged non-conventional brewers’ spent grains (BSGs) in bread-making and assess the comparative quality of bread enriched with non-fermented and lactic acid-fermented BSGs obtained from mashes brewed with starch adjuncts of buckwheat and oats. After the runoff of the first wort, unsparged non-conventional BSGs with approximately 75% moisture, acidic pH, and yield in the soluble extract above 56.6% (*w*/*w* d.m.) were used in substituting wheat flour with 5 and 15% (*w*/*w* d.m.) in bread-making recipes. The highest loaf volume value (318.68 cm^3^/100 g) was observed for 5% fermented buckwheat-BSG addition. Except for the samples with 5% fermented BSGs, specific volumes decreased. Crumb moisture was reduced by up to 22% for all samples, with this parameter related to bread weight. Bread porosity, elasticity, acidity, and overall sensory acceptability were better for fermented than non-fermented BSGs. The results proved that non-conventional BSGs with buckwheat and oats addition have the potential to be valorized in new bread assortments, and lactic acid fermentation applied to the BSGs is beneficial, even for overall sensory acceptability and quality of baked end-products. Technological, buckwheat-BSG was more convenient than oats-BSG. Further research continues to optimize and upscale Technology Readiness Levels.

## 1. Introduction

The global beer market is still growing; it was estimated at USD 706.60 billion in 2024, forecasting a yearly growth rate of 6.26% between 2024 and 2032. Additionally, beer market reports show there is a growing market for new assortments of beer brews [1], with increasing preferences for new beer tastes and flavors [2,3], making a place for beer diversification and innovation, with more attributes of health or sustainability [1,4]. With brewing advancements, a variety of so-called non-conventional beers and other specialties have been made of high quality with improved sensory (e.g., beer body, color) and nutritional functionalities (e.g., higher antioxidant capacity, gluten-free, or lower carbohydrates) [4,5]. For example, the literature discusses the processing of alternative grains as starch adjuncts, such as malted or non-malted cereals (e.g., sorghum, rice, oats, wheat, rye, corn, millet, or teff), pseudocereals (e.g., buckwheat, amaranth, and quinoa), and other non-conventional raw materials (e.g., potatoes, and cassava), combined with or instead of barley malt [4,5,6,7,8,9,10,11,12,13]. Adjunct incorporation indeed modifies the flavors and other characteristics of the beer brews, but an overall understanding of the in-depth biochemical processes is not fully understood yet [6,10,14].

But nowadays, interest also focuses on sustainability, including food waste or by-products upcycling from the beer industry, contributing as much as possible to the circular economy. For example, the most abundant brewery by-product, namely BSG, has found application as feedstock in biorefineries, nutraceuticals, cosmetics, pharmaceuticals, packaging films, and functional foods [15,16,17,18].

Generally speaking, BSG is the solid grain residue left after the wort obtained during the mashing process is separated by lautering. It consists of wet grain husks and minor parts of other layers (such as pericarp, endosperm, and aleurone). Fresh BSG regularly has around 80% moisture, and its chemical composition varies with the raw material type (e.g., barley variety, harvesting conditions, and adjuncts) and malting and mashing processes (e.g., malting and mixing times). BSG contains celluloses, hemicelluloses, lignocelluloses, gums, protein (inclusive free amino acids), and other compounds (e.g., specific polyphenols, fatty acids, monosaccharides, vitamins B, and minerals) in different ratios from treated crushed grain fractions or resulting during the conversion of different compounds (e.g., starch into sugars) in the mashing process before the wort separation. The main part of BSG’s dry matter is represented by fibers (up to 70%) and proteins (around 20–30%) [17,19,20,21,22]. Consequently, the literature cited some opportunities to valorize BSG in multiple applications, such as bioethanol, biogas, enzymes, xylitol, lactic acid, dietary fibers (e.g., lignin, cellulose, xylooligosaccharides (XOS), β-glucans), phenolic compounds, bioactive peptides (BAP), hydrolyzed proteins and BSG protein composites, volatile fatty acids, ferulic acid, etc. [15,17,18,21,22,23,24,25,26].

In food processing, BSG is used for low-fat meat products, fish burgers, fermented beverages, dairy-based products, yogurt, infant formula, meat analogs and plant-based foodstuffs, pasta, snacks, flakes, cookies, biscuits, cakes, bread, and other bakery products [16,17,19,20,22,27,28,29,30]. Notably, several technologies for obtaining valuable compounds from BSG or other BSG-enriched foods are already industrially available; others are considered emerging technologies, and most are still at Technology Readiness Levels (TRLs) of up to 4. Fresh BSG is mainly disposed of as low-cost animal feed (e.g., for cattle) or for biotechnological conversion [31,32].

Generally, a lot of opportunities to valorize grain feedstock have appeared in bread-making, such as malted and non-malted cereals and pseudocereals (whole meal, grist, flours, etc.), fermented or non-fermented, into new bakery products; these grains could be either use as a substrate for preferment or sourdough, or could be added in various percentages to replace wheat flour in bread recipes during dough preparation [33,34]. Certainly, through these new formulations, there is a double interest in developing healthier food and more sustainable alternatives due to the recognized potential of specific cereal bioactive compounds in preventing or treating various diseases [20,35,36,37]. For instance, previous studies showed that oats [38,39,40], barley [41], and buckwheat [42,43] could be used in bread-making, and they supply additional functional properties because they possess plenty of valuable nutrients [44,45,46,47]. For example, buckwheat and oats, compared to wheat, have a high content of crude fibers [48]. The anatomical part of the grain also matters in obtaining bakery products; for example, research has proved that buckwheat bran, compared to buckwheat hull, has a better effect on dough properties regarding rheological behavior [49], while buckwheat hulls and bran are better sources of antioxidants compared to buckwheat flour [50]. Regarding BSG usage in bread-making, much of the cited literature refers to the addition of conventional BSG (generally, up to 10–15%) from brewing with barley malt [16,17,20,51]. Other formulations include either fermented BSG [51,52] or conventional BSG with addition of sourdough or lactic acid bacteria [53,54] during dough preparation for bread-making.

The basic idea of our research arises from the need to develop new assortments of specialty beers with adjuncts of buckwheat and oats, and interest in high-valorized same-time craft and small brewery by-products by upcycling. The scope of the work is to exploit valuable nutrients of non-conventional BSG into functional bakery products with potential health benefits or further labeled with nutritional claims, according to [55].

The literature has analyzed the beneficial physiological effects of BSG addition in bread and other bakery foodstuffs in terms of protein enrichment [22], polyphenols [56], and dietary fiber [22]. BSG composition and properties differ depending on the brewing process, which is why they influence bread’s nutritional properties and sensory profile differently [57]. Also, Nyhan et al. (2023) determined that BSG functionality in terms of bioactive compounds increases through fermentation and enzymatic processes in the food matrix and technological performance improves in sourdough bread, as well [19].

The main aim of this experiment was to investigate the technological potential of non-conventional BSG in bread-making and assess the comparative quality of bread enriched with non-conventional BSGs obtained from mashes brewed with the addition of buckwheat and oats using different technological arrays with and without BSG fermentation. Another original aspect of this study is using unsparged BSG to make bread samples; according to the brewing technological process, this BSG is obtained after the runoff of the first wort. Our results proved that BSG generated from a brewing process in which buckwheat and oats were used as starch adjuncts has the potential to be valorized in new bread assortments. At this stage, we have tried to develop bread-making technology at TRL3; the functional properties will be tested later accordingly.

## 2. Materials and Methods

### 2.1. Materials

#### 2.1.1. Wheat Flour Type 650

Commercially available wheat flour type 650 trademarked by Băneasa Company, Bucharest, Romania was used. Wheat flour type 650 is regularly used to make white bread in domestic mass production. Its properties were analyzed following established methods according to [58,59,60]. The main characteristics of the wheat flour used in the experiment were as follows: ash—0.65% (*w*/*w*); moisture—12.5% (*w*/*w*); water absorption—52.8%; wet gluten—26% (*w*/*w*); total carbohydrates—68.2% (*w*/*w*); dietary fiber—4.5% (*w*/*w*); proteins—11.1% (*w*/*w*); total fats—1.8% (*w*/*w*).

#### 2.1.2. Bakery Yeast, Salt, and Water

Compressed bakery yeast (*Saccharomyces cerevisiae*), produced by Rompak Ltd. Pascani, Romania, was used to prepare the bread samples; it was purchased from a local store in Sibiu, Romania. The main characteristics of the bakery yeast were assessed following established methods according to [59].

The salt (sodium chloride) used in the bread recipe was iodized table salt (maximum 40 mg iodine/kg salt) purchased from the chain of stores in Sibiu, Romania, with the country of origin being Romania. Its properties were analyzed following standard methods according to [61].

Single-distilled water was used to eliminate the possible influence of water quality on the experimental results. The water subjected to distillation was sourced from the drinking water network of the city of Sibiu, Romania, and had a maximum total hardness of 5° dH. Distillation was performed with a standard laboratory water distillation unit from Boeco, Germany.

#### 2.1.3. Pilsner and Cararye Malts

Pilsner and Cararye malts were used to prepare specialty beers. Generally, Pilsner malt is made from quality barley and is the perfect base for bottom-fermented beers, while Cararye is a specialty malt made from rye through a special caramelization process to give it a caramel-like sweetness and an intense aroma of bread, coffee, dark chocolate, and dried fruit. Its quality makes it a favored option for brewers looking to add complexity and depth to their beers in styles like rye ales and lagers, rye IPAs, and various darker beers.

Pilsner and Cararye malts were procured from Weyermann^®^ Specialty Malting Company in Bamberg, Germany.

The main product specifications of Pilsner malt samples in this experimental were: extract yield—minimum 80.5% (*w*/*w*) dry basis; moisture—maximum 5.0% (*w*/*w*); color—2.5–4.5 EBC units; proteins—9.5–12% (*w*/*w*); recommended addition—up to 100%; enzyme activity—high [62]. From a sensory standpoint, it was characterized by a malty-sweet profile with subtle honey-like notes.

The main product specifications of Cararye malt were: extract yield—minimum 74% (*w*/*w*) dry basis; moisture content—maximum 6.5% (*w*/*w*); color—150–200 EBC units; recommended addition—up to 15%; enzyme activity—none [63].

#### 2.1.4. Oats (*Avena sativa*)

The whole-grain oats (Ukraine, country of origin) used in the research were bought from Solaris Plant Ltd., Bucharest, Romania. The samples’ quality was assessed using the standard methods following [58,60]. Its main characteristics were: moisture—10.6% (*w*/*w*); proteins—10% (*w*/*w*); lipids—2.0% (*w*/*w*); total carbohydrates—76% (*w*/*w*); total fibers—15% (*w*/*w*).

#### 2.1.5. Buckwheat (*Fagopyrum esculentum*)

The research used whole raw grain buckwheat, meaning uncooked grain. For simplification, throughout the text in this article, only the term “buckwheat” will be used when referring to raw buckwheat. Buckwheat grain (country of origin—Ukraine) was also purchased from Solaris Plant Ltd. Company, Bucharest, Romania, and its main characteristics were: moisture—11.9% (*w*/*w*); proteins—13.26% (*w*/*w*); lipids—3.40% (*w*/*w*); total carbohydrates—68.90% (*w*/*w*); total fibers—7% (*w*/*w*). The standard methods following [58,60] were used for quality assessment.

#### 2.1.6. LAB (Lactic Acid Bacteria) Starter Culture

Lactic acid fermentation of BSG was performed with Lactoferm^®^ Natur Yogurt, a typical dry natur yogurt LAB starter culture (NY-LAB) from Brouwland, Belgium, which contained two strains, namely, *Streptococcus thermophilus* and *Lactobacillus bulgaricus*.

#### 2.1.7. Non-Conventional BSG

Non-conventional BSG was obtained under laboratory conditions by filtering mashes to produce specialty beers based on Pilsner and Cararye malts on one side and, on another, oats or buckwheat as differentiating ingredients. Consequently, considering the raw materials used for specialty beers, this laboratory experimental study used two types of BSG by-products to be valorized in bread-making and investigate their technological peculiarities, namely, buckwheat-based BSG (BW-BSG) and oat-based BSG (O-BSG).

**Figure 1:**
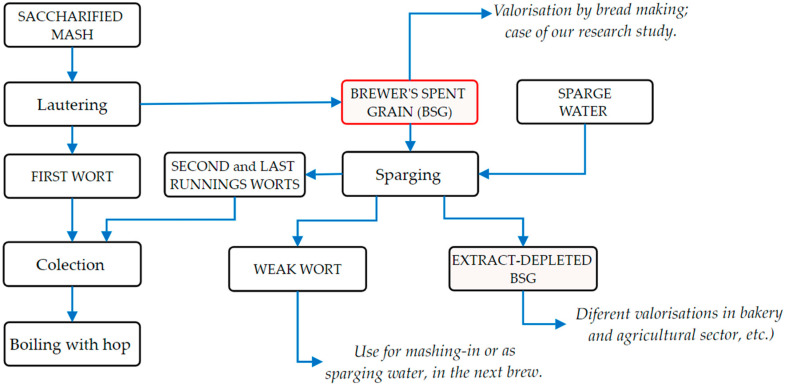
Sequence of brewing flow diagram focused on saccharified mash lautering and BSG sparging operations.

Figure 1 introduces a sequence of brewing flow diagrams focusing on saccharified mash lautering and BSG sparging operation, which is regularly applied in breweries. As is shown in the technological flow, as options for the valorizing of BSG by-product from lautering, BSG could be sparged and depleted in extracts (i.e., fermentable sugars) or discharged unsparged and valorized as it is. As depicted in Figure 1, BSG could be valorized in bread-making as an extract-depleted or unsparged BSG. In the case of this research study, BSG was removed unsparged to be upcycled into bread-making, considering that unsparged BSG could have a slightly higher nutrient content because of the remaining compounds, such as sugars, soluble proteins, and polyphenols, that did not wash out due to lack of the sparging operation.

Further details on the obtaining, summary characterization, and utilization of BSG in this study are presented in Section 2.2.

### 2.2. Methods

#### 2.2.1. BSG Obtaining and Characterization

The procedure for BSG obtaining consisted of the next steps:

1. Pilsner and Cararye malts, whole grain oats, and buckwheat were milled with a Universal laboratory Disc Mill, type DLFU, from Bühler AG, Switzerland, set to a 0.2 mm disk gap, corresponding to fine grinding.
2. The grist (with a composition of 75% *w*/*w* Pilsner malt, 10% *w*/*w* Cararye malt, and 15% *w*/*w* oats or buckwheat) was mixed with single-distilled water to a grist:water weight ratio of 1:4.5.
3. Laboratory mashing process was carried out in mashing equipment 1-Cube Mashing Bath, type R4, from 1-Cube Company, based in Havlíčkův Brod, Czech Republic, using a mashing diagram with the profile described by the following parameters: 37 °C for 10 min, 45 °C for 20 min, 62 °C for 30 min, 72 °C for 20 min, and 78 °C for 10 min; the increase of temperature was 1 °C/min; 200 rot/min was selected for the homogenization speed.
4. The saccharified mashes were lautered through qualitative folded filter paper (Ø = 320 mm, grade 597 ½ from Schleicher & Schuell Company, Germany) according to EBC Methods 4.5.1 [64] into a graduated cylinder until the amount of drained wort was very small and the lautering was considered finished. What remained on the filter paper represented the BSG, which was immediately collected, homogenized, and stored in a tightly closed container in a fridge at 4 °C until the moment of use. The corresponding non-conventional BSG, either O-BSG or BW-BSG, was used for bread-making.
5. In the particular case of fermented BSG, lactic acid-fermented BSG was produced by inoculating with 1% *w*/*w* of NY-LAB starter culture and fermenting at 42 °C for 20 h and, afterward, at 6 °C for 12 h.

The BSG was briefly characterized in the laboratory by determining the moisture content (EBC Methods 12.2) and the soluble extract (EBC Methods 12.4) [64]. At each use of the BSG sample, its moisture was determined in order to correctly establish the amount of BSG that had to be added to replace a certain percentage of the wheat flour type 650 (reported to dry matter (d.m.)) compared to the control recipe for bread-making. In addition, for both non- and fermented BSG, total titratable acidity (TTA) was determined through titrating with NaOH (sodium hydroxide) solution 0.1N in the presence of phenolphthalein 1% as the indicator and expressed in mL NaOH 1N/100g of the analyzed BSG sample.

#### 2.2.2. Bread-Making Test

The baking test consisted of dough preparation, processing, and baking and bread cooling. The dough was prepared using the straight method at room temperature. The control sample (CS) was made from 280 g wheat flour type 650 (W650), 5 g fresh bakery yeast, 4 g salt, and 160 mL water. In the experiment, several bread-making schemes were proposed, with BSG as it is or with the addition of LAB-fermented BSG instead. All ingredients put in one stage were transformed into the dough by kneading for 5 min with a laboratory mixer made by SADKIEWICZ^®^ Instruments from Bydgoszcz, Poland.

**Figure 2:**
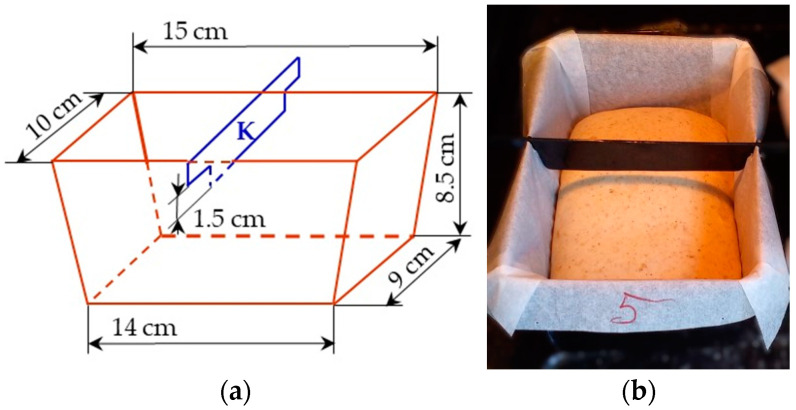
Dough leavening in the baking pan: (**a**) Drawing with the dimensions of the baking pan with knife (the K in the drawing is the knife of the baking tray); (**b**) photo of the baking pan with a dough sample that is left to leaven until it reaches the bottom of the baking pan knife.

The dough was molded and placed in a baking pan, covered with baking paper, and lightly greased with sunflower oil; the baking pan dimensions are given in Figure 2a. Then, the dough was left to leaven at 30 °C and 80 ± 5% relative humidity (RH) in the fermentation chamber of the laboratory electric oven type ESM 3710 SADKIEWICZ (made by SADKIEWICZ^®^ Instruments from Bydgoszcz, Poland), until the leavening time was achieved. The leavening duration was considered to end when the dough touched the lower part of the knife (see K from Figure 2) of the baking pan in which the dough was left to rise.

The leavened dough was baked at 210 °C for 35 min in a pre-heated oven in pre-steaming conditions (pre-steamed for 10–15 s after placing the baking pans in). After baking, the bread loaves were cooled at ambient room temperature and evaluated two hours later. All trials were performed in triplicate.

Different dough recipes were used for baking tests to investigate the technological potential of non-conventional BSG samples, either non- or lactic acid-fermented BSG, compared to CS in bread-making. For this purpose, the bread-making recipes have foreseen partial replacement of WF650 with an amount of non-conventional BSG that corresponds to 5% (*w*/*w*) and 15% (*w*/*w*) (dry basis WF650). The bread-making schemes with corresponding BSG variants applied in this study are introduced in Table 1.

Due to the variability of wet BSG obtained in laboratory conditions and keeping the dough consistency related to water absorption and the amount of added water within the recipe as raw material under control, the quantities were determined based on the calculation of technological balance in dry matter.

The calculation formulas used for determining the amount of BSG in the bread recipe, equivalent to the percentage of W650 replacement (reported d.m.), are the following:

(1) $${DM}_{RP}=\frac{RP}{100}\times {DM}_{280}=\frac{RP}{100}\times \frac{100-H_{W650}}{100}\times M_{280}\;,\;\left[g\right]$$

(2) $$M_{W650}={DM}_{RP}\times \frac{100}{100-H_{W650}}\;,\;\left[g\right]$$

(3) $$M_{BSG}={DM}_{RP}\times \frac{100}{100-H_{BSG}}\;,\;\left[g\right]$$

(4) $$W_{RP}={M_{160}-W_{BSG}=M}_{160}-\frac{H_{BSG}}{100}\times M_{BSG}\;,\;\left[g\right]$$

where:

*DM_RP_* is the amount from *DM*_280_ which must be replaced with an equivalent amount of d.m. from BSG, g;

*DM*_280_ is the amount of d.m. from *W*_280_, g;

*RP* represents the replacement percentage from *DM*_280_, % (*w*/*w*);

*H_W_*_650_ is the moisture of W650, % (*w*/*w*);

*M*_280_ is the amount of W650 in CS recipe, g;

*M_W_*_650_ is the amount of W650 to replace by BSG from CS recipe, g;

*M_BSG_* is the amount of wet BSG (having *H_BSG_*) to replace *DM_RP_* from CS recipe, g;

*H_BSG_* is the moisture of BSG, % (*w*/*w*);

*W_RP_* is the amount of water in recipe, g;

*M*_160_ is the amount of water in CS recipe, g;

*W_BSG_* is the amount of water from *M_BSG_*, g.

#### 2.2.3. Laboratory Analyses: Bread Evaluation

After the baking process and two hours of cooling at the ambient room temperature, the weight loss was determined to investigate the amount of water lost due to baking and cooling operations. The processing loss of three bread loaves per batch was measured.

The loss of weight during baking and cooling (*LB_W_*) was calculated by expressing as a percentage the difference between the weight of the dough before baking and the weight corresponding to the baked and cooled bread, related to the mass of the dough that was baked. The calculation formula used for this purpose was:

(5) $${LB}_{w}=\frac{W_{D}-W_{B}}{W_{D}}\times 100\;,\;[\%\;(w/w)]$$

where:

*W_D_* is the weight of dough before baking, g;

*W_B_* is the weight of baked and cooled bread, g.

The bread’s specific volume (*V_S_*) was determined by assessing the rapeseed volume displaced by the weighed bread sample using the method with Fornet’s device. *V_S_* was calculated using the following standard formula:

(6) $$V_{S}=\frac{V_{B}}{W_{B}}\times 100\;,\;\left[{\mathrm{cm}}^{3}/100\;g\right]$$

where:

*V_B_* is the volume of bread, equivalent to the volume of rapeseeds displaced by bread sample, cm^3^;

*W_B_* is the weight of cooled bread, g.

Bread evaluation regarding crust and crumb properties, such as bread crumb porosity and elasticity, including acidity and moisture, was conducted using Romanian standard analysis methods according to [65]; the standards of wheat bread [66] were used as a reference basis for comparison in assessment.

#### 2.2.4. Sensory Evaluation of Bread

The sensory evaluation was performed by seven assessors trained for this analysis type, who habitually consumed various kinds of bread. The trained panelists (four women and three men) were between 23 and 35 years old. They were instructed to eat unsalted white bread crumbs between each sample test to eliminate the influence of the aftertaste and to cleanse their mouths very well with drinking water (at room temperature 20–22 °C) afterward.

A simple hedonic descriptive test used a 9-point scale for the sensory evaluation. The significance of scale numbers was: 1—I surely do not like it; 2—I do not like it very much; 3—I moderately do not like it; 4—I do not like it a little; 5—I cannot say that I like it, but I would not know that I like it either; 6—I like it a little; 7—I like it moderately; 8—I like it very much; 9—Surely, I like it very much. The sensory attributes evaluated were appearance, crust color, crumb color, odor (flavor), taste, texture (mouthfeel), and overall acceptability. Panelists accepted the product if the scores were 5 or above, while scores ranging from 1 to 4 indicated rejection of the product.

#### 2.2.5. Statistical Analysis

All the measurements were done in triplicate. Analysis of variance (ANOVA) was applied to compare mean values of the samples with nF-BSG and F-BSG, respectively, at different addition levels for the two types of BSG: O-BSG and BW-BSG (see Table 1). Statistically significant differences were considered at *p* < 0.05 by the Tukey test, and MATLAB R2023b version (MathWorks, Natick, Massachusetts, USA) software was used. An experimental design was performed to identify single and combined effects of factors on the responses. The significative variables are the independent variables *x_i_*, according to which the variation of the answer will be analyzed, and the objective functions (answer functions) established (dependent variables *y_i_*) are the W650, nF-BSG, and F-BSG, following the working scheme for bread-making recipes (see Table 1). The mathematical relationship of the response y on the significant independent variables is given by the quadratic (second-degree) polynomial equation:

(7) $$y=a_{1}+a_{2}\cdot x_{1}+a_{3}{\cdot x}_{2}+a_{4}\cdot x_{3}+a_{5}\cdot x_{1}\cdot {x\;}_{2}+a_{6}\;\cdot x_{1}\cdot x_{3}+a_{7}\cdot x_{1}^{2}+a_{8}\cdot x_{2}^{2}+{\;a}_{9}\cdot x_{3}^{2}$$

where:

*y* is the dependent variable; *x*_1_ is the W650; *x*_2_ is the nF-BSG; and *x*_3_ is the F-BSG.

The selected dependent variables were the following: specific volume and weight of bread, moisture, porosity, elasticity, and total titratable acidity of bread crumb. The coefficients of each response (objective) function were calculated with MATLAB’s Curve Fitter toolbox. Afterward, the response optimization was achieved by minimizing or maximizing the objective functions whose analytical expression was previously obtained using the response surface methodology (RSM) [67].

## 3. Results and Discussion

### 3.1. BSG Characterization

In the case of the samples used in this experiment, BSG granularity cannot be controlled or adjusted by milling after BSG lautering/filtering because wet BSG is used afterward as it is, so the particle sizes were obtained by fine grinding when the grist was prepared (with a Retsch’s Laboratory Vibratory Sieve Shaker, AS 200 basic). Table 2 introduces the particle size distribution of the grist, which consisted of milled Pilsner and Cararye malts, oats, and buckwheat.

**Figure 3:**
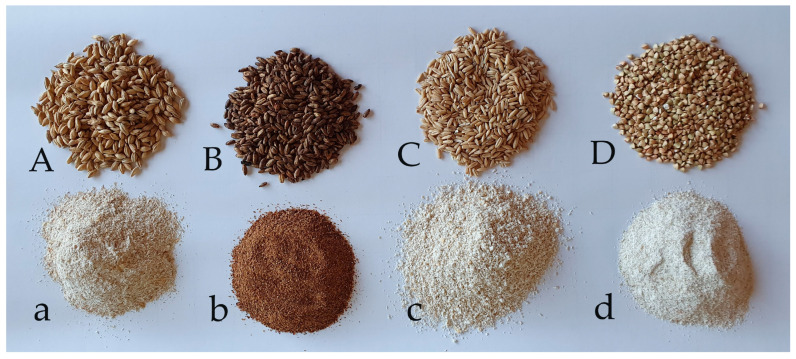
Grains used in brewing process (Pilsner malt (**A**), Cararye malt (**B**), oats (**C**), and buckwheat (**D**)) and their corresponding grist (**a**–**d**).

Figure 3 depicts the whole grains and their milled products composing the grist for the brewing process. Noteworthy, the grain grist, obtained in specified conditions, generated a non-conventional BSG with a specific texture related to its particle size.

**Figure 4:**
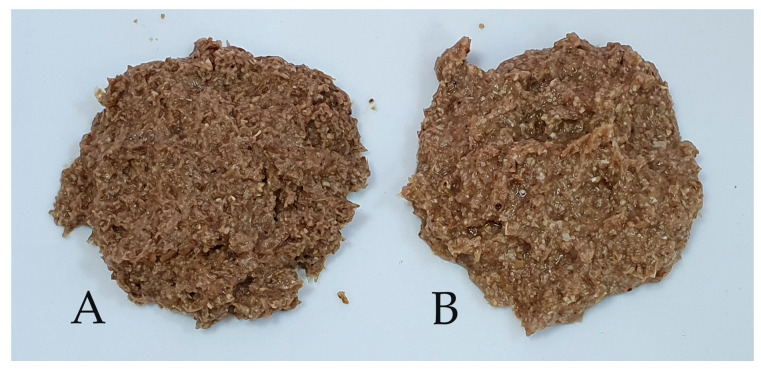
Non-conventional BSG non-fermented: oats-based BSG (**A**) and buckwheat-based BSG (**B**).

Figure 4 shows the non-conventional BSGs as fresh, non-fermented ingredients for bread-making recipes afterward. Considering that non-conventional BSG was discharged from brewing mashes prepared with Cararye malt, all BSG samples were darker than conventional BSG, which is produced exclusively from barley malt. Also, as Figure 4 shows, when oats and buckwheat were used as starch adjuncts, fresh nF-O-BSG was slightly darker than nF-BW-BSG. However, by LAB fermentation, a slight lightening in color was visually observed on non-conventional BSGs, more visible for F-BW-BSG.

O-BSG and BW-BSG characteristics were summarized in Table 3 and Table 4.

Although the same technological conditions applied to both non-conventional BSGs, the moisture content slightly differed between non-fermented O-BSG and BW-BSG. Still, the moisture content results depicted in Table 3 ranged between proximate values obtained by Jin et al. (2022) [68] when they investigated the BSG composition of various craft breweries.

Regarding yield in soluble extract, close values were determined between non-fermented, non-conventional BSGs per total, with narrow variations when the results were expressed on a dry basis; this is probably due to the comparative composition between the two types of BSG with buckwheat or oats. According to the literature [7,11,48], buckwheat is a good protein source with a better amino acid profile, carbohydrates with a lower glycemic index, and more antioxidants than oats. Also, although similar, our results indicated that nF-BW-BSG had a soluble extract yield a little bit higher than nF-O-BSG, which means a potentially slightly higher added value from a nutritional standpoint for nF-BW-BSG compared to nF-O-BSG.

Similar pH values were established between these fresh non-conventional BSGs compared to other craft breweries’ BSGs mentioned in the literature [68]. As shown in Table 4, non-fermented O-BSG had higher TTA values and lower pH than non-fermented BW-BSG, being more acidic. Obviously, through LAB fermentation, all samples of non-conventional BSG became more acidic with increased TTA and decreased pH values. However, although fresh, non-fermented O-BSG and BW-BSG started from different TTA and pH values, the growth degree, in percentage, of these parameters’ values was the same, i.e., after BSG fermentation, the increasing ratio for TTA was approximately the same at 147% for all BSGs, while for pH it was 23.90 ± 0.70%.

Concerning food safety, only potential contamination during BSG keeping before usage was considered, as a particular case of raw BSG’s upcycling, but not all food safety hazards that could occur in the craft brewery [69] or bread-making [70]. Because the specialty beer recipes were based on a mix of malted barley and rye, none of the BSGs or end products are gluten-free, even if buckwheat was added.

Notably, after filtering, the non-conventional BSG samples were packed in airtight plastic bags and stored in the fridge at 4 °C until use. The BSG preserving and handling conditions before use in bread-making allow for avoiding the samples’ contamination without raising additional food safety issues from physical and microbiological standpoints. Further experiments will discuss the more convenient preserving method in terms of costs, technological arrays, and food safety aspects related to a particular shelf life.

### 3.2. BSG Valorization in Bread-Making

In the literature, most of the BSG used in bread-making was dried before usage until reaching a moisture content lower than 7% [21,51,52,53,71], and BSG powder was tailored in the bread recipes. The literature supplies different figures regarding the percentage of BSG addition in bread-making. On the one hand, the inclusion of BSG over 10% improves the nutritional value due to an increased content of both fiber and protein [52]; on the other hand, it is challenging to introduce a higher percentage of BSG because of difficulties due to technological behavior and less sensory appeal of baked products [52]. Thus, this study proposed a comparative approach between 5% BSG, which means a slight improvement, and 15% BSG, which ensures end-products obtained with the already-proved source of fiber [52], according to Regulation EC No 1924/2006 [55].

Unsparged wet BSG (with about 75% moisture) was used in this study, which is why attention was also paid to particle size, moisture content, and food safety aspects. Because BSG moisture can be potentially variable due to brewing processing and/or storage conditions, the percentage of its addition was calculated relative to W650 on a dry basis. Based on the results obtained applying the Equations (1)–(4), the quantities of wet BSG and added water as they are used in the bread-making recipe were determined and the results are introduced in Table 5.

Wet BSG was used as such, resulting from brewing and filtering with no additional grinding applied since the process would be difficult at this stage. Attention was paid to fine-milling grains before brewing (see Table 2); in that way, from an organoleptic standpoint, wet BSG would not have a coarse texture when incorporated into the dough during its preparation.

Previous research highlighted that BSG addition during dough preparation influences the physical properties and rheological behavior of dough, especially when BSG inclusion into a recipe is over 10% (*w*/*w*); namely, water absorption increases, probably due to fiber intake in the dough, as well as dough development time [52], while dough strength and viscosity decrease [17]. However, the rheological behavior of dough was assessed only organoleptically (visual, tactile, etc.), not with rheological equipment, in this study. The baking test method in a pan was chosen to avoid certain differences between samples due to dough consistency.

As shown in Table 6, during dough proofing, the leavening duration varied between doughs depending on BSG types, probably due to their particular composition. For non-fermented BW-BSGs, proofing times were higher than nF-O-BSGs, while for fermented BSGs, the trends were the opposite. Proofing times increased for all fermented oat-BSG samples, while lower proofing times of fermented buckwheat-BSG samples were registered (with a 14–24% ratio than nF-BW-BSGs).

Regarding the technological aspects of bread-making, Table 6 introduces the processing losses (of baking and cooling) for all variants during bread-making. The figures indicated lower bread weight losses for nF-O-BSGs and nF-1BW compared to CS, while for all fermented non-conventional BSG samples, these processing losses were above those for the control sample.

### 3.3. Bread Analysis

**Figure 5:**
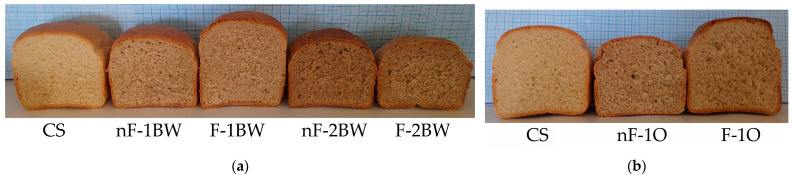
Baking tests: (**a**) Breads with buckwheat-BSGs (nF—non-fermented and F—fermented) compared to control sample (CS); (**b**) breads with oats-BSGs (nF—non-fermented and F—fermented) compared to control sample (CS).

The results of baking tests are presented in Figure 5. At first glance, the characteristics of bread loaves showed significant changes for almost all the sensory attributes when non-conventional BSGs were used as ingredients, with notable differences between LAB-fermented and non-fermented BSG feedstock, and depending on the percentage of BSG addition to the bread recipes.

All bread samples with BSG addition were colored more than the control sample in bread crust and crumb color. Color browning could be explained on the one hand by BSG color itself (see Figure 4). On the other hand, unsparged BSG comes with more fermentable sugar and amino acids that could be additionally involved in Maillard’s reaction during baking with more colored end-products. As observed in Figure 5, the color of the crust and breadcrumb shifted towards more intense but pleasant shades of brown and brick red with the increase in BSG addition to bread. Still, the bread samples obtained with the fermented variants of BSG presented lighter colors than non-fermented BSGs at the same addition level.

Appearance and breadcrumb characteristics improved through lactic fermentation of BSGs compared to non-fermented BSG samples. The smell and taste of the BSG breads were pleasant and more intense than the control samples. Concerning the texture in terms of the mouthfeel of the bread samples, it was observed that the partial substitution of wheat flour (W650) with F-BSGs has improved the mouthfeel for all additional levels of F-BSG.

Baking tests revealed that the best sensory characteristics, loaf volume, and breadcrumb properties were obtained for the 5% addition of fermented buckwheat-based BSG. Overall, in our experiment, buckwheat-based BSG was a more convenient choice compared to oats-based BSG when upcycling BSG in bread-making.

Also, lactic acid-fermented BSGs have conferred superior properties to the bread compared to the non-fermented variants. Our results agree with data from the literature that considered the beneficial effects of lactic acid fermentation on bread production with BSG [19,51,52,54]. The proximate composition of the enriched samples in BSG was not determined, but according to the literature [52,57], BSG comes with additional content of protein, crude fiber, and ash.

**Figure 6:**
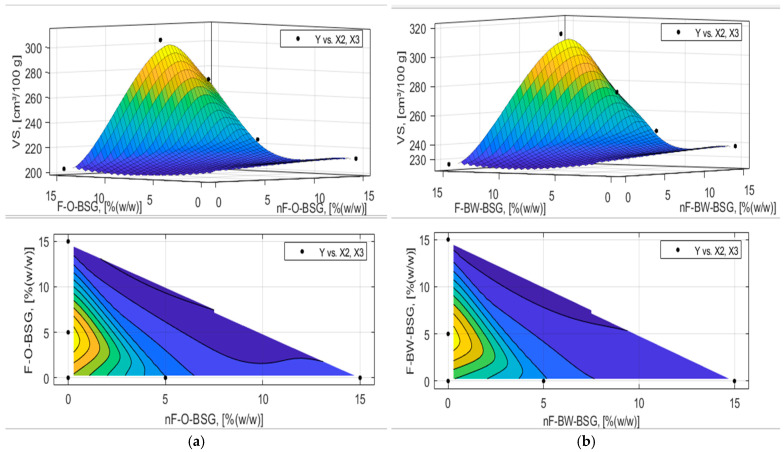
Three-dimensional surface plot and contour plot presenting the influence of nF-BSG and F-BSG on specific volume (V_S_, [cm^3^/100 g]) of bread loaves made with: (**a**) O-BSG; (**b**) BW-BSG.

**Figure 7:**
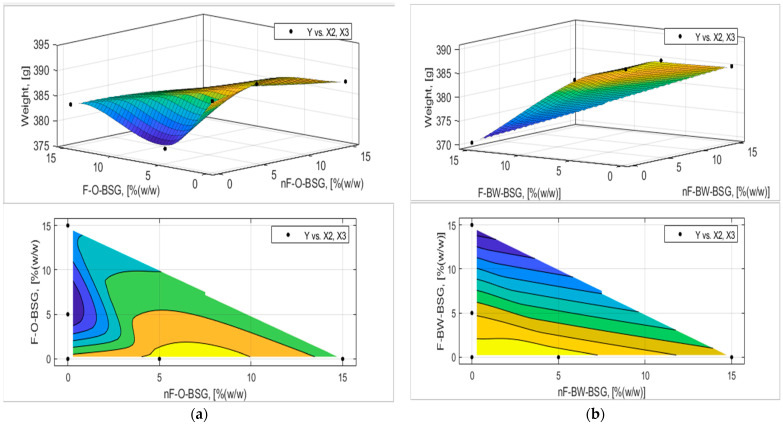
Three-dimensional surface plot and contour plot presenting the influence of nF-BSG and F-BSG on weight (W_B_, [g]) of bread loaves made with: (**a**) O-BSG; (**b**) BW-BSG.

**Figure 8:**
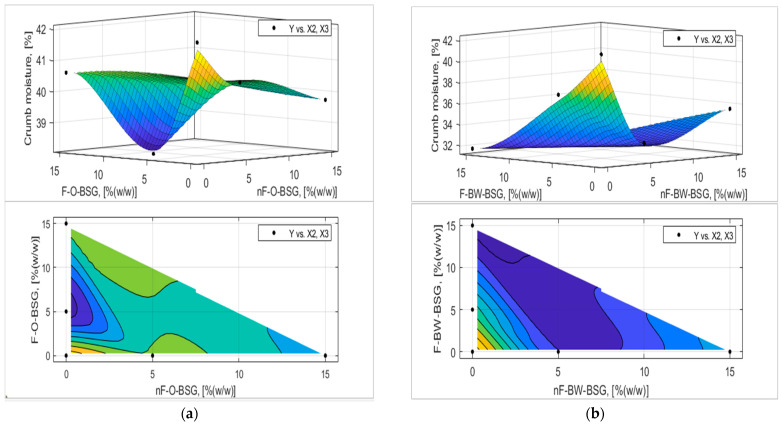
Three-dimensional surface plot and contour plot presenting the influence of nF-BSG and F-BSG on crumb moisture (H_C_, [% (*w*/*w*)]) of bread loaves made with: (**a**) O-BSG; (**b**) BW-BSG.

**Figure 9:**
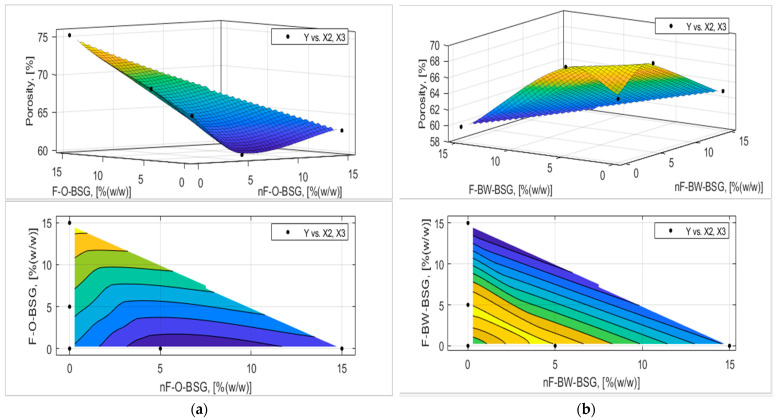
Three-dimensional surface plot and contour plot presenting the influence of nF-BSG and F-BSG on crumb porosity (P_C_, [% (*v*/*v*)]) of bread loaves made with: (**a**) O-BSG; (**b**) BW-BSG.

**Figure 10:**
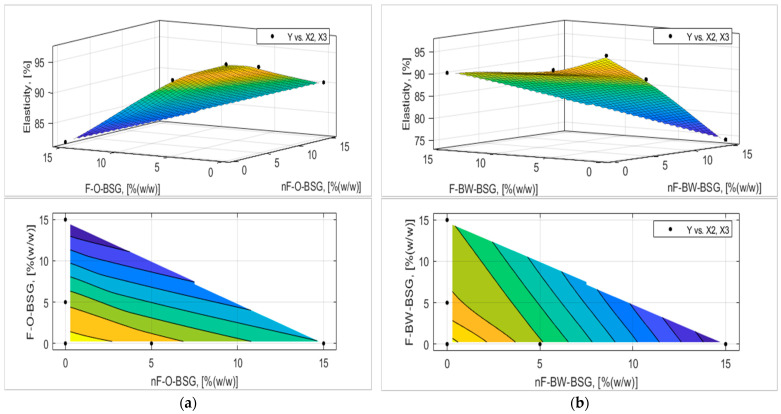
Three-dimensional surface plot and contour plot presenting the influence of nF-BSG and F-BSG on breadcrumb elasticity (E_C_, [% (*v*/*v*)]) of bread loaves made with: (**a**) O-BSG; (**b**) BW-BSG.

**Figure 11:**
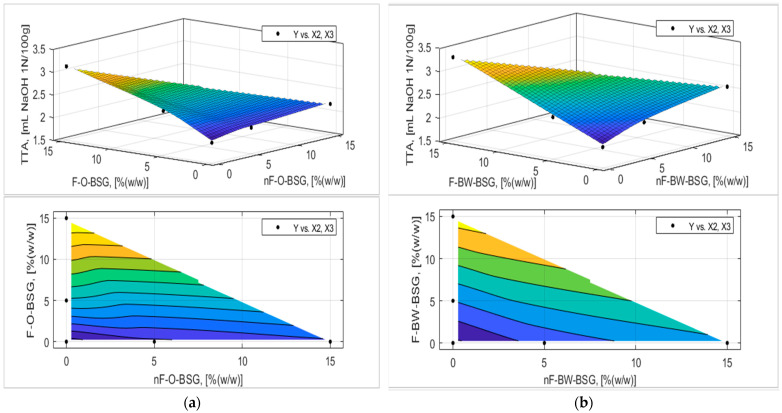
Three-dimensional surface plot and contour plot presenting the influence of nF-BSG and F-BSG on total titratable acidity (TTA, [mL NaOH 1N/100 g]) of breadcrumb for samples made with: (**a**) O-BSG; (**b**) BW-BSG.

The analytic expression of the response functions was determined using experimental data and expression (7) proposed in Section 2.2.5 with MATLAB’s Curve Fitter toolbox. The adequacy of the analytical expressions of the response functions was verified with the Fischer test, and the regression coefficients were tested, eliminating those that were not interesting. The final equations of the answer functions are presented in Table 7 and Table 8, and the graphical representation of the response functions are shown in Figure 6, Figure 7, Figure 8, Figure 9, Figure 10 and Figure 11.

The graphs in Figure 6 show that for both types of BSGs, i.e., based on oats and buckwheat, the maximum specific volume was obtained in their fermented variant for the replacement percentage of 5% (*w*/*w*) d.m. of W650 in the recipe. Both values of these specific volumes were higher (V_S_F-1BW_ = 318.68 ± 1.14 cm³/100 g; V_S_F-1O_ = 309.58 ± 1.04 cm³/100 g) compared to that of the control sample, namely V_S_CS_ = 279.96 ± 1.05 cm³/100 g.

Optimal values were estimated through the three-dimensional surface plot. The mathematical model corresponds to the experimental results, but the values calculated by the model are slightly lower than those effectively determined. For example, for bread enriched with 5% (*w*/*w*) fermented buckwheat-BSG, a specific volume of V_S_F-1BW_ = 318.68 ± 1.14 cm³/100 g was assessed, while using modelling a max of 315.247 cm³/100 g was estimated for a percent of 4.53% (*w*/*w*) F-BW-BSG added. In addition, all bread samples with BW-BSG as an ingredient presented a higher specific volume compared to similar samples, but based on O-BSG. This finding shows that F-BW-BSGs are preferable to oats-based ones (F-O-BSG). Thus, considering this bread quality parameter, BW-BSG has a greater potential for technological exploitation than O-BSG in these pre-set conditions.

Referring to the weight of the bread loaves, Figure 7 indicates that the samples using the non-fermented variants of both types of BSG at replacement levels 1 and 2 had higher masses than the fermented BSGs; nF-BSG-bread loaves had similar values to the control sample (=389.34 ± 0.96 g) from a weight standpoint. However, bread samples with nF-O-BSG had higher masses (W_B_nF-1O_ = 390.87 ± 1.01 g; W_B_nF-2O_ = 387.67 ± 1.15 g), compared to those with nF-BW-BSG (W_B_nF-1BW_ = 389.54 ± 1.26 g; W_B_nF-2BW_ = 385.05 ± 1.40 g). These data are correlated with the results obtained for processing losses, i.e., LBw (% (*w*/*w*) (see Table 6) and positively correlated with breadcrumb moisture (see Figure 8). For example, as shown in Figure 7 and Figure 8, the weight of the bread loaves with oat-BSG is higher and correlated with increased breadcrumb moisture values of the corresponding samples.

Crumb moisture was reduced by up to 22% for all samples.

The experimental results and those established on the mathematical model (see Figure 9 and Figure 10) showed that breadcrumb porosity and elasticity are improved for the bread samples by adding oat-BSG compared to buckwheat-BSG. Overall, bread porosity and elasticity were better for fermented than non-fermented BSGs. For example, as it follows from Figure 9, the maximum porosity obtained by optimization based on the regression model was achieved at an addition of 14.43% fermented oats-BSG (P_max_ = 74.68% compared with P_CS_ = 66.09%). Still, the best values for bread elasticity were registered for the control sample; maximum values of the response surface based on the regression model varied between nF- and F-BSGs (see Figure 10).

Bread acidity significantly increased by adding BSGs, more for buckwheat-BSGs than oat-BSGs, and, obviously, higher for fermented than non-fermented BSGs (see Figure 11). The total titratable acidity of samples was positively affected (*p* < 0.05) by the addition of higher levels of O-BSG and BW-BSG, in the non-fermented or fermented variants.

The TTA values for all bread samples were higher than the TTA of the control sample, specifically 1.69 ± 0.01 mL NaOH 1N/100 g. The highest crumb acidity value was assessed for F-2BW, approximately double compared with CS. In interpreting the results for TTA, it should be noted that the BSGs added to the bread-making formulations, depending on the starch adjuncts used, had acidities that differed from each other by 10% in the non-fermented variant and 6.5% in the fermented variant.

However, if TTA is correlated with the sensory analysis results and the main quality characteristics of the bread samples, it can be stated that lactic fermentation has a positive effect on the utilization of BSG as an ingredient in bread production, with a significant influence on flavor, taste, and color.

Overall, the lactic acid-fermented BSG positively enhanced bread quality. In this sense, previous reports provide relevant details regarding the efficacy of lactic acid fermentation in cereal-based products fortified with BSG, as in the case of bread [51,54,56,71]. The positive changes were highlighted primarily by the increase in the loaf bread volume for which fermented BSG was used as an ingredient. Presumptive exopolysaccharides produced during lactic acid fermentation can probably simulate gluten properties and act as hydrocolloids in the dough. On the other hand, incorporating 15% of BSGs leads to bread loaves with lower volume and high crumb density, which generated lower scores for the appearance and overall acceptability of the respective bread samples. In working hypotheses of higher BSG amount addition, the results could be explained by decreasing the gluten percentage in dough composition during bread-making by these high-BSG recipes. Further investigations will be conducted to assess a potential mechanism or explain these attempts regarding non-conventional BSGs used in bread-making

### 3.4. Sensory Outcomes of the BSG-Enriched Breads

**Figure 12:**
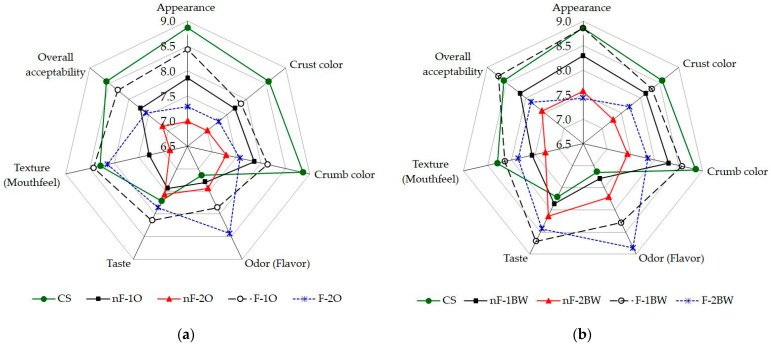
Spider web diagrams of sensory evaluation of all bread loaves: (**a**) Bread samples with nF- and F-O-BSG incorporation at different levels; (**b**) Bread samples with nF- and F-BW-BSG incorporation at different levels. Legend: CS—control sample; O—oats; BW—buckwheat; nF—non-fermented; F—fermented; 1—substitution of 5% (*w*/*w*) dry basis W650; 2—substitution of 15% (*w*/*w*) of W650 as d.m.

Regarding sensory evaluation, the bread loaves were analyzed using a hedonic descriptive statistical method as described in Section 2.2.4 and Section 2.2.5; the score for each sensory attribute was calculated as the mean ± SD of the ratings provided by the trained panelists (*n* = 7). The obtained results are represented graphically in the form of spider web diagrams, that is, Figure 12a for bread made with variants of O-BSG and Figure 12b in the case of using variants of BW-BSG for bread-making. Figure 12 reveals significant changes for almost all the sensory attributes considered for evaluation compared to the control sample.

The willingness of consumers to purchase bread is strongly affected by bread color. This preliminary research on the substitution of wheat flour with wet non-conventional BSG, as is, and fermented, reported that the color of crust and breadcrumb shifts towards more intense but acceptable pleasant, in agreement with other studies [56]. The bread samples obtained with the fermented variants of BSGs presented lighter colors for these sensory characteristics than nF-BSG. The most significant influence in this direction was observed for the samples prepared with F-BW-BSG; the highest color score was achieved by the F-1BW bread sample (crust color score: 8.29 ± 0.49; crumb color score: 8.57 ± 0.53) and the lowest score was obtained by the nF-2O test (crust color score: 7.00 ± 0.00; crumb color score: 7.29 ± 0.49). However, the darkening of the bread samples would not have a negative influence on the assessors’ perception; on the contrary, in correlation with the flavor and texture of these bread samples, it makes it possible for them to be included in the category of nutritious bread obtained from wholemeal flours, with or without added of bran or other sources of fibers and nutritional compounds.

As observed in Figure 12, all the samples with F-BSG, especially those with a higher degree of substitution, developed a more intense specific flavor and taste and, consequently, were rated better by the tasters. This finding is in accordance with the results of the research carried out by Neylon et al. (2021) and Waters et al. (2012) [51,54], in which the positive effect of the lactic fermentation of BSG on the quality of the bread, also, was highlighted. Regarding this aspect, the samples with F-BW-BSG obtained a higher score (8.71 ± 0.49 for the taste of F-1BW and 8.86 ± 0.38 for the flavor of F-2BW) compared to those containing F-O-BSG (8.14 ± 0.38 for taste of F-1O and 8.43 ± 0.53 for flavor of F-2O). In addition, the bread loaves containing nF- and F-BW-BSG were rated higher in terms of flavor and taste than those of 100% (*w*/*w*) W650 breads (an increase in line with the level of addition of BSG). In contrast, both bread samples containing nF-O-BSG received a lower score than the CS sample in terms of taste.

It emerged that the mouthfeel during chewing the crumb does not adhere to the teeth’ surface; at the same time, it is not crumbly. The texture was better for the samples with F-O-BSG (F-1O: 8.43 ± 0.53) but not much compared to those with F-BW-BSG (F-1BW: 8.14 ± 0.3). In previous reports [52,53,54,71], the addition of BSG had similar effects on the texture of bread samples, which can be explained through the difference in chemical composition between oats and buckwheat and by the influence of lactic fermentation on the fermentative substrate. A reduction in crumb hardness was also observed, the textural attribute being also essential in the sensory evaluation of bread.

The best results were obtained for the replacement of 5% (*w*/*w*) d.m. of W650 with F-BW-BSG, but up to 15% (*w*/*w*) F-BSG can be an acceptable limit in the bread recipe based on trained panelists’ preference; preliminary evaluations provided quite good scores for overall acceptability (see Figure 12). Furthermore, this sensory tasting analysis confirms the potential of BSG as a valuable food ingredient in its wet and fermented variants, which can open new approaches to production planning at the micro-technological level, in tandem with brewing and bakery.

Thus, from a sensory point of view, the addition of BSG for bread-making in studied variants in this research is beneficial to a certain extent for all the considered sensory attributes. Additionally, the tasters indicated a degree of appreciation of the sensory attributes by assigning numbers on the rating scale between 9 and 6; numbers that have attached the expression “I like it” for the description. This suggests that the use of O-BSG and BW-BSG in the non-fermented variants, but especially in the fermented variants, presents potential for the bakery industry, in terms of consumer acceptability. These results on non-conventional BSGs are in agreement with findings of other authors that used in their experiments mainly conventional BSGs [51,54].

## 4. Conclusions

There are the preliminary results of a holistic study on circularity approach from brewing to bread-making, where non-conventional BSGs from craft breweries could be upcycled in new assortments of functional, enriched breads. Compared with other studies, in this study were used non-conventional BSGs resulting from specialty beer processing with the addition of unmalted oats and buckwheat as starch adjuncts; another particularity of this research consisted of using unsparged raw BSGs instead of dried ones.

Considering the ingredients from which BSG is generated in the brewery and its processing method for incorporation into the bread recipe, the obtained results provide the recommendation of using the lactic acid-fermented variant of BSG and the one based on buckwheat. In addition, the formulation of cereal mixtures for BSG preparation and their processing method must be considered, these results being in accordance with previous studies [50,52,68,69]. However, even if within this work a composition analysis was not performed, others’ results justify the usage of BSG as an alternative feedstock for bread-making in the sense that there is potential for the development of functional breads [11,51,52,68] labeled as sources of fibers (see [55]) at least. Also, bread enriched with non-conventional BSGs received a good response as regards consumer acceptability.

Based on the outcomes of this work and considering sustainable synergy between the interdisciplinary fields of brewing and bread-making, further opportunities and research directions will focus on optimizing and TRL upscaling for upcycling non-conventional BSG. Afterward, the functionality of baked products will be checked for functional or nutritional claims.

## Figures and Tables

**Table 1:** The working scheme for bread-making recipes.

Main Ingredients	Variants of Bread-Making								
	CS	nF-1O	nF-2O	F-1O	F-2O	nF-1BW	nF-2BW	F-1BW	F-2BW
W650, [% (*w*/*w*)]	100	95	85	95	85	95	85	95	85
nF-BSG, [% (*w*/*w*)]	0	5	15	0	0	5	15	0	0
F-BSG, [% (*w*/*w*)]	0	0	0	5	15	0	0	5	15

Legend: CS—control sample; W650—wheat flour type 650; O—oats; BW—buckwheat; BSG—brewers’ spent grain; nF—non-fermented; F—fermented; 1—substitution of 5% (*w*/*w*) dry basis W650; 2—substitution of 15% (*w*/*w*) of W650 as d.m.

**Table 2:** The particle size distribution of Pilsner and Cararye malts, oats, and buckwheat milled with a Universal laboratory Disc Mill set to a 0.2 mm disk gap.

Number of Sieve (Separated Fractions’ Name)	Mesh Width (mm)	Separated Fractions on Sieve (% *m*/*m*)			
		*Pilsner* Malt	Cararye Malt	Oats	Buckwheat
1 (Husks)	1.250	1.98 ± 0.01	0.05 ± 0.001	12.33 ± 0.34	0.81 ± 0.01
2 (Coarse grists I)	0.630	12.07 ± 0.17	12.16 ± 0.19	43.48 ± 1.00	8.27 ± 0.26
3 (Coarse grists II)	0.400	28.32 ± 0.56	37.14 ± 0.81	12.95 ± 0.21	21.66 ± 0.63
4 (Fine grists I)	0.315	15.37 ± 0.31	16.03 ± 0.40	5.37 ± 0.06	13.03 ± 0.29
5 (Fine grists II)	0.250	13.03 ± 0.14	8.73 ± 0.19	6.17 ± 0.11	8.64 ± 0.32
6 (Fine grists III)	0.160	24.09 ± 0.19	11.79 ± 0.22	13.23 ± 0.31	11.27 ± 0.17
Bottom (Flour)	-	5.14 ± 0.13	14.10 ± 0.28	6.47 ± 0.14	36.32 ± 0.72

Values mean values of triplicate determinations (*n* = 3) ± standard deviation.

**Table 3:** Moisture and yield in soluble extract of non-fermented O-BSG and BW-BSG.

Type of Non-Conventional BSG	Characteristics		
	Moisture, [% (*w*/*w*)]	Yield in Soluble Extract,	
		[% (*w*/*w*) as Is]	[% (*w*/*w*) d.m.]
nF-O-BSG	74.8 ± 0.89	14.26 ± 0.17	56.62 ± 0.68
nF-BW-BSG	75.1 ± 1.02	14.27 ± 0.19	57.30 ± 0.84

Legend: O-BSG—oat-based BSG; BW-BSG—buckwheat-based BSG; nF—non-fermented. Values represent the mean ± standard deviation (*n* = 3).

**Table 4:** Total titratable acidity and pH of non- and lactic acid-fermented BSGs.

Characteristics	nF-O-BSG	nF-BW-BSG	F-O-BSG	F-BW-BSG
Total titratable acidity (TTA), [mL NaOH 1N/100 g BSG as is]	4.20 ± 0.06	3.78 ± 0.06	10.41 ± 0.12	9.73 ± 0.08
pH	5.73 ± 0.08	5.82 ± 0.09	4.32 ± 0.05	4.47 ± 0.04

Legend: O-BSG—oat-based BSG; BW-BSG—buckwheat-based BSG; nF—non-fermented; F—fermented. Values represent the mean ± standard deviation (*n* = 3).

**Table 5:** The recipes for bread-making for all bread samples.

Main Ingredients	Variants of Bread-Making								
	CS	nF-1O	nF-2O	F-1O	F-2O	nF-1BW	nF-2BW	F-1BW	F-2BW
W650, [g]	280	266	238	266	238	266	238	266	238
nF-BSG, [g]	0	48.61	145.83	0	0	49.20	147.60	0	0
H_nF-BSG_, [% (*w*/*w*)]	-	74.80	74.80	-	-	75.10	75.10	-	-
F-BSG, [g]	0	0	0	46.76	140.27	0	0	48.42	145.26
H_F-BSG_, [% (*w*/*w*)]	-	-	-	73.80	73.80	-	-	74.70	74.70
Water, [g]	160	123.64	50.92	125.49	56.48	123.05	49.15	123.83	51.49
Yeast, [g]	5	5	5	5	5	5	5	5	5
Salt, [g]	4	4	4	4	4	4	4	4	4
Percentage of BSG relative to W650 amount in recipe, [% (*w*/*w*)]	0	18.27	61.27	17.58	58.94	18.50	62.02	18.20	61.03

Legend: CS—control sample; W650—wheat flour type 650; O—oats; BW—buckwheat; BSG—brewers’ spent grain; nF—non-fermented; F—fermented; H-_BSG_—BSG’s moisture; 1—substitution of 5% (*w*/*w*) dry basis W650; 2—substitution of 15% (*w*/*w*) of W650 as d.m.

**Table 6:** The leavening duration of the dough and the loss baking for all variants of bread-making process.

Variants of Bread-Making								
CS	nF-1O	nF-2O	F-1O	F-2O	nF-1BW	nF-2BW	F-1BW	F-2BW
Leavening duration, [min]								
30 ± 0.20 ^a^	37 ± 0.25 ^b^	42 ± 0.22 ^c^	58 ± 0.32 ^d^	50 ± 0.30 ^e^	54 ± 0.33 ^a^	57 ± 0.40 ^a^	41 ± 0.33 ^a^	49 ± 0.23 ^a^
Weight loss, *LB_w_* [% (*w*/*w*)]								
12.26 ± 0.09 ^a^	11.36 ± 0.09 ^b^	11.86 ± 0.09^c^	13.58 ± 0.12 ^d^	12.50 ± 0.11 ^a^	11.63 ± 0.11 ^a^	12.44 ± 0.08 ^a^	13.08 ± 0.13 ^a^	14.53 ± 0.07 ^a^

Legend: CS—control sample; O—oats; BW—buckwheat; nF—non-fermented; F—fermented; 1—substitution of 5% (*w*/*w*) dry basis W650; 2—substitution of 15% (*w*/*w*) of W650 as d.m. Each value in the table is the mean ± standard deviation (*n* = 3). Values sharing different letters within a line are significantly different (*p* < 0.05).

**Table 7:** The equations of response functions considering the significant independent variables for bread loaves made with O-BSG.

Dependent Variables (*y*) and Regression Equation for the Response Function
Specific volume, [cm³/100 g] $y=0.9993+0.9708\cdot x_{1}+0.9834\cdot x_{2}+0.9718\cdot x_{3}-0.019\cdot \;x_{1}\cdot x_{2}+0.1508\cdot \;x_{1}\cdot x_{3}+0.0182\cdot \;x_{1}^{2}+0.4297\cdot \;x_{2}^{2}-0.971\;x_{3}^{2}$; $reznorm=1.6734\cdot 10^{-7}$
Weight of bread, [g] $y=0.9993+0.9712\cdot x_{1}+0.9806\cdot x_{2}+0.9821\cdot x_{3}+0.0633\cdot \;x_{1}\cdot x_{2}+0.0267\cdot \;x_{1}\cdot x_{3}+0.0291\cdot \;x_{1}^{2}-0.0076\cdot \;x_{2}^{2}+0.1818\cdot \;x_{3}^{2}$; $reznorm=6.1249\cdot 10^{-8}$
Crumb moisture, [%] $y=0.9993+0.9696\cdot x_{1}+0.9799\cdot x_{2}+0.9803\cdot x_{3}-0.0148\cdot \;x_{1}\cdot x_{2}-0.022\cdot \;x_{1}\cdot x_{3}-0.0056\cdot \;x_{1}^{2}+0.0042\cdot \;x_{2}^{2}+0.0489\;x_{3}^{2}$; $reznorm=6.8959\cdot 10^{-8}$
Porosity, [%] $y=0.9993+0.9697\cdot x_{1}+0.9805\cdot x_{2}+0.98\cdot x_{3}-0.0221\cdot \;x_{1}\cdot x_{2}-0.0003\cdot \;x_{1}\cdot x_{3}-0.0032\cdot \;x_{1}^{2}+0.0714\cdot \;x_{2}^{2}+0.0028\cdot \;x_{3}^{2}$; $reznorm=6.7402\cdot 10^{-8}$
Elasticity, [%] $y=0.9993+0.9699\cdot x_{1}+0.9798\cdot x_{2}+0.9796\cdot x_{3}-0.0032\cdot \;x_{1}\cdot x_{2}-0.0054\cdot \;x_{1}\cdot x_{3}-0.0001\cdot \;x_{1}^{2}-0.0141\cdot \;x_{2}^{2}-0.0384\;x_{3}^{2}$; $reznorm=7.9014\cdot 10^{-8}$
Total titratable acidity (TTA), [mL NaOH 1N/100 g] $y=0.9993+0.9695\cdot x_{1}+0.9797\cdot x_{2}+0.9797\cdot x_{3}-0.019\cdot \;x_{1}\cdot x_{2}-0.0181\cdot \;x_{1}\cdot x_{3}-0.0096\cdot \;x_{1}^{2}-0.0104\cdot \;x_{2}^{2}-0.0113\;x_{3}^{2}$; $reznorm=7.5637\cdot 10^{-8}$

**Table 8:** The equations of response functions considering the significant independent variables for bread loaves made with BW-BSG.

Dependent Variables (*y*) and Regression Equation for the Response Function
Specific volume, [cm³/100 g] $y=0.9993+0.9715\cdot x_{1}+0.982\cdot x_{2}+0.9719\cdot x_{3}-0.0348\cdot \;x_{1}\cdot x_{2}+0.1703\cdot \;x_{1}\cdot x_{3}+0.0182\cdot \;x_{1}^{2}+0.2325\cdot \;x_{2}^{2}-0.9772\;x_{3\;}^{2}$; $reznorm=1.8655\cdot 10^{-7}$
Weight of bread, [g] $y=0.9993+0.9714\cdot x_{1}+0.9806\cdot x_{2}+0.9802\cdot x_{3}+0.0602\cdot \;x_{1}\cdot x_{2}+0.0543\cdot \;x_{1}\cdot x_{3}+0.0291\cdot \;x_{1}^{2}-0.0015\cdot \;x_{2}^{2}-0.0328\;x_{3\;}^{2}$; $reznorm=7.9466\cdot 10^{-8}$
Crumb moisture, [%] $y=0.9993+0.9694\cdot x_{1}+0.9807\cdot x_{2}+0.9802\cdot x_{3}-0.0359\cdot \;x_{1}\cdot x_{2}-0.0207\cdot \;x_{1}\cdot x_{3}-0.0056\cdot \;x_{1}^{2}+0.105\cdot \;x_{2}^{2}+0.0402\;x_{3}^{2}\;$; $reznorm=6.083\cdot 10^{-8}$
Porosity, [%] $y=0.9993+0.9701\cdot x_{1}+0.9793\cdot x_{2}+0.9791\cdot x_{3}+0.0038\cdot \;x_{1}\cdot x_{2}+0.0048\cdot \;x_{1}\cdot x_{3}-0.0032\cdot \;x_{1}^{2}-0.0753\cdot \;x_{2}^{2}-0.0948\cdot \;x_{3}^{2}$; $reznorm=9.0775\cdot 10^{-8}$
Elasticity, [%] $y=0.9993+0.9698\cdot x_{1}+0.9802\cdot x_{2}+0.9802\cdot x_{3}-0.0126\cdot \;x_{1}\cdot x_{2}-0.0107\cdot \;x_{1}\cdot x_{3}-0.0001\cdot \;x_{1}^{2}+0.0319\cdot \;x_{2}^{2}+0.0289\;x_{3}^{2}$; $reznorm=6.846\cdot 10^{-8}$
Total titratable acidity (TTA), [mL NaOH 1N/100 g] $y=0.9993+0.9695\cdot x_{1}+0.9797\cdot x_{2}+0.9797\cdot x_{3}-0.01186\cdot \;x_{1}\cdot x_{2}-0.0184\cdot \;x_{1}\cdot x_{3}-0.0096\cdot \;x_{1}^{2}-0.011\cdot \;x_{2}^{2}-0.0088\;x_{3}^{2}$; $reznorm=7.5475\cdot 10^{-8}$

## Data Availability

The original contributions presented in the study are included in the article, further inquiries can be directed to the corresponding authors.
